# Technology-Themed Persecutory and Related Presentations of Delusions in the Digital Age: Narrative Review

**DOI:** 10.2196/102927

**Published:** 2026-08-14

**Authors:** Syed Ali Bokhari, Abdelaziz A Osman, Muhanad Elnoor, Mohamed A Alnor, Syed Fahad Javaid

**Affiliations:** 1 Al Amal Psychiatric Hospital, Emirates Health Services, Al Aweer, Dubai, United Arab Emirates; 2 University of Medical Sciences and Technology, Khartoum, Sudan; 3 Departmeng of Psychiatry, Al Ain Hospital, Al Ain, Abu Dhabi, United Arab Emirates; 4 Department of Psychiatry, College of Medicine and Health Sciences, United Arab Emirates University, Sheikh Khalifa Bin Zayed Street, Al Ain, Abu Dhabi, United Arab Emirates, 971 503979565

**Keywords:** persecutory delusions, technology, internet, Truman Show delusion, influencing machine, cyber-paranoia, pathoplasticity, psychosis, social media, AI, algorithmic delusion, conspiracy ideation

## Abstract

**Background:**

Persecutory delusions have long mirrored prevailing cultural and technological concerns. Beliefs involving implanted devices, internet surveillance, hacked smartphones, algorithmic targeting, and AI-mediated control are increasingly visible in contemporary psychosis. However, we identified no prior review dedicated specifically to synthesizing technology-themed delusional content across historical and contemporary clinical contexts.

**Objective:**

This narrative review aimed to trace the historical evolution of technology-themed delusions, characterize their contemporary clinical phenomenology, examine relevant neurocognitive and sociocultural mechanisms, and discuss implications for psychiatric assessment and intervention.

**Methods:**

PubMed/MEDLINE, Scopus, and APA PsycInfo were searched without date restrictions, supplemented by backward citation tracking and targeted identification of historical primary sources. Eligible sources included original research, case reports and case series, reviews, and relevant conceptual or historical publications addressing delusional content involving technology. A narrative synthesis approach informed by the Scale for the Assessment of Narrative Review Articles (SANRA) framework was used. The final search was completed on May 1, 2026.

**Results:**

Forty-three sources were included in the narrative synthesis, comprising 9 case reports or case series, 14 empirical studies, 8 reviews, and 12 historical or conceptual sources. Technology-themed delusions appeared as pathoplastic variants of enduring persecutory, control, and referential motifs, clustering into 5 overlapping categories: surveillance and hacking beliefs, implant and control delusions, Truman Show–type broadcast phenomena, social media–specific referential ideas, and emerging algorithmic and AI-mediated themes. In the largest contemporary cohort (228 patients with psychosis), technology-themed delusions were described by 104 of the 201 (51.7%) patients with delusional thought content, and the odds of technology-themed delusions rose by approximately 15% per admission year (odds ratio 1.15, 95% CI 1.01-1.31; *P*=.04). Cognitive models of persecutory ideation, the aberrant salience hypothesis, technological unfamiliarity, digital immersion, and wider sociocultural narratives all appeared relevant to these presentations. Pandemic-era conspiracy material, including 5G and vaccine-microchip narratives, further illustrated how culturally available technological explanations may scaffold delusional elaboration in vulnerable individuals.

**Conclusions:**

Technology-themed delusions represent contemporary expressions of enduring delusional motifs shaped by digital culture. Their assessment requires psychiatric expertise combined with sufficient technological literacy to distinguish proportionate privacy concerns, overvalued conspiracy beliefs, and fixed delusional convictions. Future research should use prospective cohort designs, cross-cultural comparisons, and targeted evaluation of cognitive behavioral and digital literacy–informed interventions, while monitoring emerging AI-mediated phenomena.

## Introduction

Persecutory delusions, the most prevalent subtype of delusional ideation, are characterized by beliefs about being harassed, threatened, or conspired against [1,2]. Although the broad categories of delusion, such as persecutory, grandiose, and referential, remain stable across cultures and historical periods, their specific thematic content is profoundly influenced by the prevailing sociocultural and technological environment [3,4]. These categories are not sharply separable: persecutory beliefs lie on a continuum with milder and more prevalent phenomena such as ideas of reference and social-evaluative concern, and they frequently co-occur with referential, control, and grandiose content [5]. The present review therefore considers persecutory delusions together with these related nonpersecutory forms wherever the source material allows. This pathoplastic principle has been demonstrated repeatedly: religious themes dominated in earlier centuries, political themes tracked geopolitical events, and technological themes have emerged alongside each new wave of innovation [6].

In the digital era, a growing body of case reports and cohort studies has documented the incorporation of internet surveillance, microchip implantation, algorithmic manipulation, and social media–mediated persecution into delusional frameworks [7,8]. The “device in the brain” delusion, wherein an individual believes a technological device has been implanted to monitor or control their cognition, represents 1 particularly striking manifestation. However, it sits within a wider spectrum that includes beliefs about hacked phones, Truman Show–type broadcast delusions [9], and algorithmically mediated control. As technologies become more ubiquitous and less intuitively understood, the boundary between plausible concern and delusional conviction can become harder to assess clinically [10].

Although related discussions exist within broader reviews of culture, psychosis, and social media [11,12], to our knowledge, after database and citation searching, no prior review has specifically synthesized technology-themed delusional content across historical and contemporary clinical contexts. An updated and dedicated synthesis is warranted less because the core structure of delusions has changed, since conviction, preoccupation, and distress remain relatively stable across eras, than because the pace, opacity, and ubiquity of contemporary technologies have rapidly enlarged the content available for delusional elaboration and blurred the boundary between proportionate concern and pathological conviction in ways that bear directly on present-day assessment.

Against this background, the present review addresses a focused question: how have technology-themed delusions and related nonpersecutory phenomena evolved across historical and contemporary contexts, and what do their phenomenology and mechanisms imply for clinical assessment in the digital age? To answer this, the review aims to (1) trace the historical evolution of technology-themed delusional content; (2) characterize its contemporary clinical phenomenology across persecutory and related referential, control, and grandiose themes; (3) examine contributing neurocognitive, emotional, and sociocultural mechanisms; and (4) discuss implications for psychiatric assessment and intervention.

## Methods

### Study Design and Rationale

This review adopts a narrative synthesis approach informed by the Scale for the Assessment of Narrative Review Articles (SANRA) framework [13]. A narrative rather than systematic design was chosen because the existing literature on technology-themed delusional content is characterized by methodological heterogeneity, a predominance of case-level reports, and limited standardization of terminology, precluding meaningful quantitative pooling or formal risk-of-bias assessment. The review protocol was not preregistered; narrative reviews fall outside the scope of PROSPERO (International Prospective Register of Systematic Reviews), which registers only reviews with a systematic methodology and a health-related outcome.

### Search Strategy

Database-adapted versions of a common conceptual search were run in PubMed/MEDLINE, Scopus, and APA PsycInfo, complemented by backward citation tracking of the included articles and by targeted location of historical primary sources (eg, archive [14] for the Haslam [15] monograph and the Jay [16] monograph on the air loom case). The conceptual search combined a named-phrase block (established terms in the literature, including “technology delusion,” “internet delusion,” “internet-related psychosis,” “influencing machine,” “Truman Show delusion,” “cyber-paranoia,” “microchip delusion,” “electronic chip implant,” “social media psychosis,” “AI psychosis,” “AI delusion,” “chatbot delusion,” and “large language model [LLM] psychosis”) with a concept block built from technology and AI terminology AND psychosis terminology AND a relevance filter (content, cultural, pathoplastic*, historical, persecutory, or “case report”); the 2 blocks were combined with OR. The full search strings used in each database are reported in Multimedia Appendix 1. No date restrictions were imposed. Non-English records were considered when the title and abstract or an accessible full-text translation allowed adequate assessment of relevance and content; full-text translation was not undertaken as a separate formal procedure. The final search was run on May 1, 2026. Google Scholar was used informally to locate cited works and for backward citation tracking but was not used as a formal indexed-database search.

### Eligibility Criteria and Study Selection

Original research, case reports, reviews, and relevant conceptual or historical sources were eligible for inclusion, provided they addressed delusional content incorporating technology. Sources concerned solely with technology-based interventions for psychosis, digital phenotyping, or screen-time epidemiology without reference to delusional content were excluded. Records were screened based on title and abstract by 2 authors (ME and SAB) for relevance; full texts were assessed where eligibility was uncertain. Disagreements were resolved through discussion with a senior author (AAO). This screening process was used to identify thematically relevant material for narrative synthesis and was not conducted as a prospectively logged systematic-review screening cascade.

### Data Synthesis

After screening and supplementary citation tracking, 43 sources were included in the final narrative synthesis: 9 case reports or case series, 14 empirical studies, 8 reviews, and 12 historical or conceptual sources. The full breakdown is provided in Multimedia Appendix 2. The completed SANRA checklist is provided in Checklist 1 [13]. Included sources were grouped by historical period, technology substrate, delusional theme, and explanatory framework. Categories were derived inductively: 2 authors (ME and SAB) grouped each source by its dominant technology substrate and delusional theme; provisional groupings were compared and refined iteratively through discussion until a stable set of categories emerged, and disagreements were resolved with the senior author (AAO). This process produced the 5-category typology presented in the *Results* section, with historically influential publications prioritized alongside clinically informative contemporary reports. Consistent with a narrative-review methodology, no formal risk-of-bias tool was applied, and PRISMA (Preferred Reporting Items for Systematic Reviews and Meta-Analyses)-style records-identified, records-screened, and records-excluded counts were not retained. The limitations inherent in this approach, including the nonsystematic search strategy and the potential for selection bias, are addressed in the *Discussion* section.

## Results

### Historical Evolution

One of the earliest detailed accounts of a technology-themed delusion dates to 1810, when Haslam documented James Tilly Matthews, who believed he was being controlled by an “air loom” operated through magnetically charged gases [15,16]. The seminal 1919 paper by Tausk [17], cited here alongside its later English translation, described patients who attributed thoughts, movements, and bodily sensations to mysterious “influencing machines” that incorporated the most advanced technology available at the time while retaining an inexplicable, quasi-mystical quality [18]. These foundational observations established that delusional content draws upon the technological imaginaries of each era.

The introduction of broadcast media produced measurable shifts in delusional themes. Škodlar et al [3], analyzing over a century of Slovenian psychiatric records (1881-2000), found a statistically significant increase in delusions of influence and control following the adoption of radio and television [3]. Cannon and Kramer [6] observed analogous patterns in American psychiatric records spanning 1913 to 1999, with technology-themed delusions centered on radio, television, telephone, and radar increasing across the century. These longitudinal analyses established the empirical basis for the pathoplasticity hypothesis in relation to technological change [6].

An internet-themed delusion was reported in the late 1990s [19], and similar presentations were subsequently documented across multiple countries (Table 1). A large contemporary retrospective single-center cohort study of 228 adults with psychosis receiving treatment in a thought disorders-intensive outpatient program between 2016 and 2024 found that technology delusions were described by 104 of the 201 (51.7%) patients with delusional thought content [8]. Logistic regression showed that the odds of technology-related delusions increased by approximately 15% per year across the study period, with social media platforms, smartphones, and algorithms featuring prominently. Patients described beliefs about hacked devices, encoded online messages, and thought transmission via text messages [8].

**Table 1.** Chronological overview of selected influential or clinically relevant sources on technology-themed delusions^a^.

Author (Year)	Country	Design	Sample, n	Key findings
Haslam [15], 1810	United Kingdom	Historical case account	1	First documented “influencing machine” delusion: the “air loom”
Tausk and Feigenbaum [18], 1992; Tausk [17], 1919	Austria	Clinical-theoretical paper	Multiple clinical examples	Described influencing machines as projections of the body; coined the concept
Škodlar et al [3], 2008	Slovenia	Retrospective cohort	120 (1881‐2000)	Significant increase in technical delusions after radio and television adoption
Cannon and Kramer [6], 2012	United States	Retrospective cohort	Hospital records (1913‐1999)	Technology-themed delusions increased across the 20th century; 76.5% (78/102) persecutory
Tan et al [19], 1997	Canada	Case report	1	Early internet-themed persecutory delusion in schizophrenia
Bell et al [7], 2005	United Kingdom	Case series	4	Internet influenced delusion etiology, form, and content; illustrated CBT^b^-based therapeutic approach
Lerner et al [20], 2006	Israel	Case report	2	Internet delusions as modified persecution and broadcasting delusions
Hirjak and Fuchs [21], 2010	Germany	Case series	3	Phenomenological analysis of “technical alien control” delusions; pathoplasticity confirmed
Nitzan et al [22], 2011	Israel	Case series	3	Internet-related psychosis in previously well individuals; loneliness and digital illiteracy as risk factors
Gold and Gold [9], 2012	United States	Case series	5	Described the Truman Show delusion; proposed the “Suspicion System” model
Mason et al [23], 2014	United Kingdom	Cross-sectional	181	Developed the Cyber-Paranoia and Fear Scale; cyber-paranoia distinct from general paranoia
Burns et al [8], 2025	United States	Retrospective cohort	228 (2016‐2024)	Technology delusions in 104 of the 201 (51.7%) patients with delusional content; approximately 15% annual increase in odds (odds ratio 1.15, 95% CI 1.01-1.31); social media themes prominent
Yang and Crespi [11], 2025	Canada	Systematic review	Multiple studies	Broad social-media review; proposed mechanisms by which online environments may amplify delusion-like processes

a Studies are listed chronologically. Sources comprise original research, case reports, reviews, and selected conceptual or historical sources directly relevant to the topic.

b CBT: cognitive-behavioral therapy.

Several recurrent clinical observations merit emphasis. Multiple authors have observed that patients with no prior familiarity with the internet or computers nonetheless incorporated internet themes into their delusional content [20,24], suggesting that cultural salience, not only direct personal experience, may influence the adoption of technological themes. Another study identified shared vulnerability factors among its patients, including loneliness or a recent loss, relative technological inexperience, and an absence of prior psychiatric history [22]. A 2005 study noted that internet-themed delusions appeared only after the internet achieved sufficient cultural prominence, implying a threshold of societal awareness before technological concepts enter the delusional repertoire [7]. One study went so far as to propose that electronic chip-implant delusions might represent a novel culture-bound syndrome [25], although subsequent authors have generally favored viewing them as pathoplastic variants of established delusional forms [4,20].

### Clinical Phenomenology and Typology

Synthesizing the available literature, technology-themed delusions can be classified into 5 overlapping categories (Table 2): surveillance and hacking belief, implant and control delusions, broadcast and referential delusions (including the Truman Show delusion), social media–specific referential ideas, and emerging AI and algorithmic delusions. In a recent Burns cohort, surveillance and hacking beliefs were the most common presentations [8], while Gold and Gold [9] provided the most detailed characterization of broadcast-type presentations.

**Table 2.** Proposed typology of technology-themed delusions with clinical examples^a^.

Category	Description	Clinical examples
Surveillance and hacking	Belief that personal devices are compromised for monitoring purposes	Phone or Wi-Fi hacked by government; spyware installed to record conversations through the device microphone; cameras embedded in walls
Implant and control	Belief that a device has been implanted in the body to control cognition or behavior	Microchip in brain transmitting or monitoring thoughts; tracking device implanted during surgery; Neuralink-type control
Broadcast and referential (Truman Show–type)	Belief that one’s life is being filmed, broadcast, or displayed for an audience	Life is a reality show; being watched by millions; everyone around is an actor
Social media–specific	Persecutory or referential ideas centered on specific platforms or features	Encoded messages in Instagram posts; Spotify playlists containing personalized messages; Facebook profiles sending coded signals
Algorithmic and AI	Belief about being monitored or controlled by algorithms or AI	Social media algorithm reading thoughts; AI directing life events; chatbot conspiracies

a This typology is heuristic and intended for descriptive clinical use rather than a validated classification system. Categories are not mutually exclusive; patients may present with features of multiple categories simultaneously. Illustrative sources informing the examples include Bell et al [7], Burns et al [8], Gold and Gold [9], Lerner et al [20], Eytan et al [25], Hirjak and Fuchs [21], and Yildirim Budak and Yazici Karabulut [26].

Hirjak and Fuchs [21] proposed that these delusions may involve more than classical themes simply being renamed in technological language [21]. They identified 4 properties of modern technology that may render it particularly salient to altered self-experience in psychosis: concealed mechanisms, dissolution of spatial boundaries, ease of producing sensory illusions, and reification of mental processes. The gangstalking phenomenon, analyzed by Lustig et al [27] through multimodal discourse analysis of YouTube evidence videos, further illustrates the interplay between technology, persecutory belief systems, and the self-reinforcing dynamics of digital self-documentation; because those materials were not drawn from clinically diagnosed samples, they are best interpreted as sociocultural rather than psychiatric evidence and should not be used to infer clinical prevalence [27].

The Truman Show delusion warrants particular attention as a paradigmatic technology-era presentation. Gold and Gold [9] first described this phenomenon as the conviction that one’s life is being continuously filmed and broadcast. In 5 reported cases, patients explicitly referenced the 1998 film, and the authors argued that the syndrome represents a culturally shaped variant of persecutory, grandiose, and referential themes. Their “Suspicion System” formulation, a cognitive mechanism for social threat detection that becomes dysregulated in psychosis, provides a complementary explanatory framework to the cognitive model by Freeman. A later adolescent case during the COVID-19 period, linked to intensive webcam-based online education, illustrates how prolonged digital immersion can become incorporated into Truman-type psychopathology in vulnerable individuals [26].

The COVID-19 pandemic further illustrated how a shared societal event can rapidly enlarge the pool of culturally available technological explanations on which delusional content may draw. More broadly, the pandemic amplified technology-related conspiracy narratives in public discourse, including beliefs linking 5G networks to viral transmission and beliefs about microchip implantation through vaccination [28,29]. Delusion-proneness, a trait phenotype that exists on a continuum in the general population, has been shown to predict endorsement of such COVID-19 conspiracy beliefs and resistance to belief updating [30], suggesting that this enlargement of the shared stock of culturally available technological explanations may carry particular clinical salience for individuals at risk of psychosis [31]. This broadening is consistent with broader cultural accounts of how shared narratives shape psychopathology [32].

### Neurocognitive and Sociocultural Mechanisms

The cognitive model by Freeman [33,34] conceptualizes persecutory delusions as threat beliefs arising from a search for meaning in anomalous or emotionally significant experiences, maintained by reasoning biases (particularly the “jumping to conclusions” bias), anxiety, safety-seeking behaviors, and low self-confidence. Emotion is integral to this account rather than incidental. Anxiety and catastrophic worry bring implausible threat ideas to mind and sustain them, and cognitive-behavioral interventions that reduce worry reduce persecutory delusions [35], while depression supplies the negative affect and negative self-evaluation that help maintain persecutory conviction [36]. These emotional processes interact with negative-self schemas (beliefs about the self as vulnerable or inadequate) and negative-other schemas (beliefs about others as hostile or malicious), which are central components of the cognitive model and dispose the individual to read ambiguous technological events as personally threatening [33]. The uncertainty and unfamiliarity that surround rapidly changing technologies can heighten exactly this anxious, ruminative, and mistrustful appraisal, particularly in less digitally experienced individuals.

Within this framework, the technological environment supplies both the raw material for anomalous experiences, such as device glitches and algorithmically personalized content, and the explanatory schemas through which those experiences are interpreted as threatening. Bell et al [7] observed that the internet features in delusions in 2 principal ways: as an explanation for anomalous experiences and as a medium of perceived persecution [7]. Burns et al [8] extended this observation within their cohort, noting that misinterpretation of routine technological processes, such as a Wi-Fi router resetting, an application logo changing, or text messages disappearing over time, could trigger persecutory fears, particularly in digitally inexperienced users [8]. The aberrant salience hypothesis [37] provides a complementary neurobiological framework, suggesting that dysregulated dopaminergic signaling leads to the inappropriate attribution of significance to neutral stimuli; the omnipresence and complexity of modern technology greatly expand the landscape of potentially salient stimuli available for incorporation into persecutory narratives [37].

**Figure 1.**
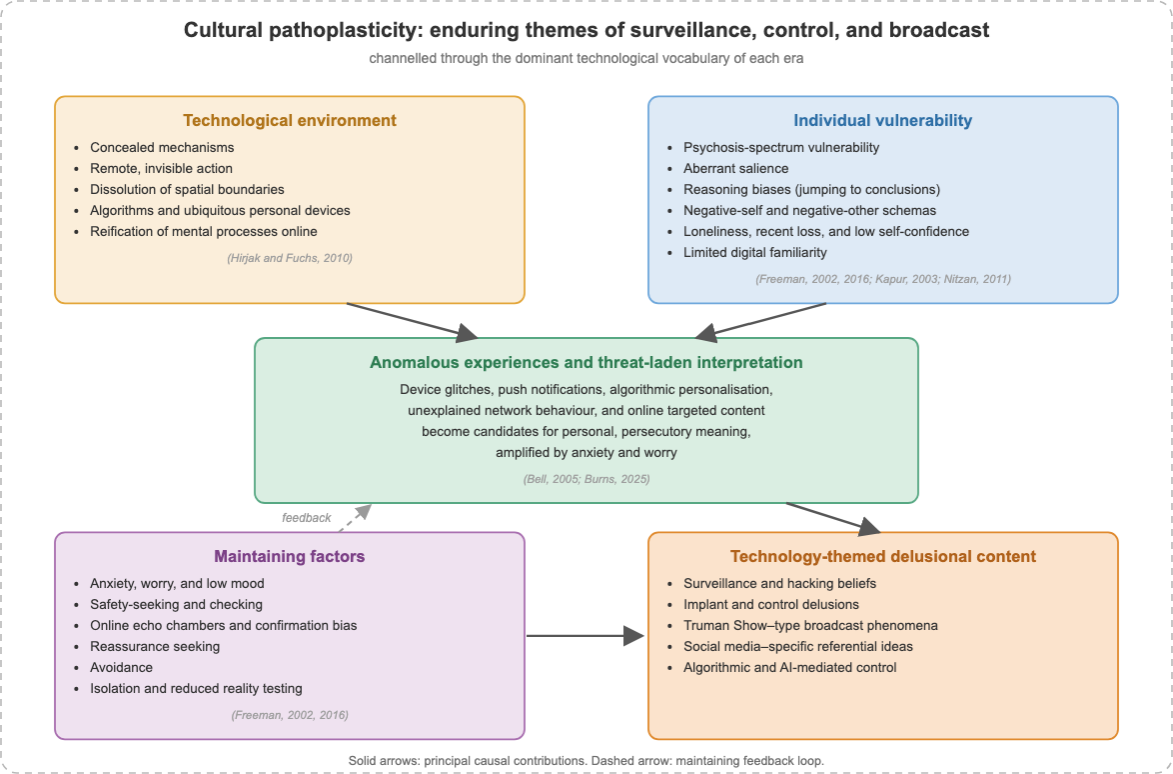
Conceptual framework for the formation and maintenance of technology-themed delusions. Cultural pathoplasticity channels enduring themes of surveillance, control, and broadcast through contemporary technological vocabularies. The model highlights interactions among the technological environment, individual vulnerability factors (including negative-self and negative-other schemas), anomalous experiences, and their threat-laden interpretation and maintaining factors such as anxiety, worry, low mood, checking, reassurance seeking, and online reinforcement [7,8,21,22,33,34,37].

Sociocultural factors further shape these presentations. Declining institutional trust, mass surveillance revelations, science fiction narratives, and the COVID-19 period have all contributed ready-made templates for technology-themed persecution [32]. Mason et al [23] demonstrated that cyber-paranoia constitutes a distinct dimension of threat perception, with technological unfamiliarity predicting higher levels of such fears [23]. Yang and Crespi [11], in a broader systematic review of social media use and disorders of the social brain, proposed mechanisms by which disembodied online interaction may amplify delusion-like processes, and although not specific to persecutory delusions, their framework remains relevant to technology-themed psychosis [11]. Figure 1 summarizes the interaction among the technological environment, individual vulnerability factors, and sociocultural context in shaping technology-themed delusional content.

The continuum model of paranoia provides an important contextual framework and is supported by the nonclinical study of 1202 participants by Freeman et al [5], which found a continuous distribution of paranoid thoughts without evidence of a distinct subpopulation. Technology-themed delusional content exists on a spectrum from widespread concerns about digital privacy, through subclinical conspiracy ideation, to fully formed delusional beliefs. The clinical challenge lies in accurately positioning an individual’s beliefs on this continuum and distinguishing proportionate concern from pathological conviction.

### Diagnostic and Therapeutic Implications

Technology-themed delusions pose distinctive diagnostic challenges. The rapid pace of technological development means that beliefs that might once have been dismissed as implausible can overlap with genuine concerns about privacy, surveillance, or algorithmic influence. Consistent with this shift, the *Diagnostic and Statistical Manual of Mental Disorders*, Fifth Edition, Text Revision (*DSM-5-TR*) no longer requires delusions in delusional disorder to be nonbizarre [1], underscoring the limited value of a rigid bizarre vs nonbizarre distinction. Clinicians must also differentiate technology-themed delusions from overvalued conspiracy beliefs or culturally shared but false ideas in nonclinical populations [10]. This technological judgment is not a substitute for traditional delusion assessment but should be integrated with it: the interviewer weighs bizarreness alongside the degree of conviction, preoccupation, and incorrigibility or fixity with which a belief is held [38], and sufficient technological literacy then helps to judge whether a given concern reflects an accurate observation, a misinterpretation, or a fixed delusional attribution [8].

Therapeutically, cognitive-behavioral approaches targeting persecutory beliefs have the strongest evidence base [34]. Bell et al [7] described the use of collaborative empiricism in an internet-themed delusion, using the internet itself to test and ultimately disconfirm the belief [7]. Emerging digital interventions, including smartphone-based ecological momentary assessment and intervention for delusional distress [39], represent a promising but still preliminary frontier. Psychoeducation targeting digital literacy may also be worth exploring for at-risk individuals, given observed associations between technological unfamiliarity and cyber-paranoia or internet-related psychosis [22,23]. Routine clinical inquiry about internet use, device-related concerns, and social media habits may be clinically useful when assessing persecutory beliefs [8,22].

## Discussion

### Summary of Findings

This review synthesizes over 2 centuries of evidence on technology-themed delusions. Several conclusions emerge. First, the reviewed literature supports the pathoplasticity of delusional content: enduring themes of surveillance, control, and broadcast are repeatedly re-expressed through the dominant technological vocabulary of a given era, from Matthews’ air loom to contemporary smartphone and algorithmic narratives [16]. Second, technology-themed delusions may involve more than superficial relabeling. Hirjak and Fuchs [21] proposed that concealed mechanisms, remote action, and the apparent objectification of mental processes make modern technologies especially resonant with altered self-experience in psychosis [21]. Yang and Crespi [11] extended this reasoning to the social media domain [11]. Third, although persecutory content predominates in the literature, technology-themed delusions are not confined to it: the reviewed material also spans control and influence phenomena, from the influencing machine to implant and device-control beliefs; referential content, such as social media–specific ideas of reference; and grandiose presentations, most clearly, the Truman Show delusion, which has been characterized as a culturally shaped variant of persecutory, grandiose, and referential themes. This breadth is consistent with treating persecutory delusions and related nonpersecutory forms along a single continuum. Across 2 centuries, the reviewed material is therefore consistent with a structural reading of pathoplasticity in which a small number of enduring delusional motifs (surveillance, control, broadcast, persecution, referentiality) recur, while the technological vocabulary in which they are expressed is replaced wave by wave; the central question raised by the recent literature is whether contemporary technologies merely supply a new vocabulary or whether they additionally alter the form of the delusional experience itself.

### Algorithmic and AI-Mediated Delusions: An Emerging Frontier

Early commentaries, media-informed clinical concerns, and emerging survey data have raised concerns about a particular emerging frontier concerning delusional phenomena structured around 3 overlapping but distinguishable AI substrates: recommendation algorithms that curate the content a user encounters, conversational chatbots that engage the user in dialogue, and generative AI systems that produce novel text, image, or audio output on demand. These substrates carry somewhat different psychopathological resonances. Recommendation-algorithm phenomena map most naturally onto surveillance and persecutory beliefs, since contemporary algorithms achieve a precision of personalization that is experientially indistinguishable from targeted monitoring. Burns et al [8] reported such themes among the most contemporary content in their cohort, with patients describing the impression that social media platforms or smartphones were reading their thoughts or selecting content to harass them [8]. Conversational chatbots, by contrast, articulate more directly with broadcast and referential phenomenology, since their natural-language responsiveness invites attribution of intentional agency to a system that has none, which dovetails with the aberrant salience model of psychosis [37]; a separate clinical literature has begun to document delusional experiences arising or being reinforced through extended dialogue with LLMs, often via the model’s affirming, highly responsive, and context-sensitive outputs that may validate distorted beliefs [40]. Recent empirical work supports this concern: a cross-sectional survey of 1003 young adults found that those at elevated psychosis risk were significantly more likely to engage in intensive generative AI use, to ascribe human-like roles (companion, friend, therapist, or romantic partner) to chatbot interactions, and to report delusion-related content within those interactions [41].

Building on this, Flathers et al [42] have proposed a clinically useful 4-part functional typology in which LLM systems may act as catalyst (precipitating new psychotic symptoms in previously well individuals), amplifier (worsening preexisting symptoms), coauthor (participating in the development of harmful narratives through extended dialogue), or object (becoming the focus of delusional beliefs themselves) [42]. Synthesizing these strands, Hudon and Stip [43] frame AI psychosis not as a new diagnostic entity but as a way of understanding how sustained, anthropomorphic engagement with conversational systems may trigger, amplify, or reshape psychotic experience in vulnerable users, situated at the intersection of individual predisposition and an increasingly immersive algorithmic environment [43].

This reading fits the central argument of the present review: the AI substrate supplies a new and unusually interactive technological vocabulary for enduring surveillance, control, and referential motifs, while its responsiveness and apparent agency may, in some cases, move beyond supplying content to coshaping the delusional narrative itself. Generative AI systems further blur the boundary between user and machine output by producing contextually responsive yet ungrounded content with the texture of personal communication. Clinicians should be alert to the possibility that future presentations may increasingly involve generative AI substrates, including beliefs about being personally addressed by an algorithm, being trained on by an AI system, having one’s thoughts read by a model, or being targeted via image-generation tools. Routine inquiry about chatbot use may be reasonable when assessing persecutory, referential, or AI-themed beliefs.

### The Continuum Between Conspiracy Ideation and Frank Delusion

The boundary between socially shared conspiracy belief and clinical delusion is a recurring theme across the reviewed literature and was sharpened during the COVID-19 pandemic by the widespread circulation of 5G and vaccine-implantation narratives. Forensic work by Pierre emphasizes the inadequacy of a binary delusional vs nondelusional categorization for beliefs that draw on widely available misinformation, since the same content can be held with nonclinical conviction in the general population and with delusional intensity in individuals with psychotic disorders [10]. Empirical evidence supports this continuum view: delusion-proneness as a trait phenotype predicts both endorsement of pandemic-related conspiracy beliefs and resistance to belief updating [30], and the same trait predicts vaccination-related behavioral outcomes mediated by conspiracy ideation [31]. From a clinical perspective, the relevant question is rarely whether a belief is internally implausible but rather whether it is held with delusional conviction, occupies a disproportionate mental life, drives functional impairment, and is associated with the broader phenomenology of psychosis. Preexisting cognitive biases, low trust in institutions, and digital information environments that selectively reinforce confirmation can all push trait-level conspiracy ideation toward the delusional end of the continuum in vulnerable individuals. These distinctions may also have downstream relevance for forensic assessment, although the present review synthesizes clinical and conceptual literature rather than forensic samples, so any forensic application would require dedicated study [10,27].

### Implications for Clinical Assessment

The reviewed literature supports several practical recommendations for clinical assessment. First, routine intake should include direct inquiry about device use, social media platforms, online interactions, and beliefs about algorithms or surveillance, in the same way that questions about substance use and recent stressors are standard [8,22]. Second, the interviewer’s own technological literacy substantially affects assessment quality. A clinician who does not understand how recommendation algorithms operate may find it difficult to distinguish a patient’s accurate observation of personalized content from a delusional misattribution and may either overpathologize legitimate concern or dismiss material that warrants clinical attention. Third, the bizarre vs nonbizarre distinction has limited operational value in this domain because contemporary technologies legitimately enable forms of monitoring, geolocation, and inference about mental states that would have appeared bizarre a decade ago [1]. Clinicians should weight conviction, preoccupation, distress, functional impact, and the wider phenomenology of psychosis rather than the surface plausibility of the belief. Fourth, clinicians working with patients holding technology-themed delusional content should anticipate that culturally available conspiracy material will provide ongoing scaffolding for delusional elaboration, particularly during periods of public-health or geopolitical disruption [28]. Fifth, the assessment should attend to the patient’s premorbid relationship with technology, since both digital unfamiliarity and intense, isolated digital engagement appear to confer vulnerability through different mechanisms [22,23].

### Therapeutic Considerations

Therapeutic considerations are similarly evolving. Cognitive behavioral therapy for psychosis, which targets persecutory beliefs through identification and modification of unhelpful reasoning biases and through behavioral experiments to test threat predictions, retains the strongest evidence base and is directly applicable to technology-themed presentations [33,34]. A collaborative-empiricism case reported by Bell et al [7] demonstrates how the technological substrate of a delusion can itself be deployed therapeutically: the patient and the clinician used the internet together to test specific predictions of the belief, with the technology that had supplied the delusional material now supplying the disconfirming evidence. Smartphone-based ecological momentary assessment and intervention represent a methodological frontier with face validity for content that is itself digitally mediated, although outcome evidence remains preliminary and is largely derived from feasibility-stage trials [39]. Adjunctive psychoeducation focused on digital and algorithmic literacy may be particularly relevant for patients in whom technological unfamiliarity contributed to the onset, although direct trial evidence in the technology-themed-delusion subgroup is currently absent [22,23]. Pharmacological treatment follows standard schizophrenia-spectrum protocols, and there is currently no evidence base to suggest that technology-themed content alters the response to antipsychotic medication. However, because patients with surveillance- and monitoring-themed delusional content frequently distrust digital infrastructure, adherence strategies that minimize perceived surveillance, including long-acting injectable formulations chosen for clinical reasons rather than electronic adherence-monitoring tools, deserve specific consideration in this subgroup.

### Strengths and Limitations

Strengths of this review include its historical breadth, spanning over 2 centuries of clinical and conceptual material from the 1810 account of James Tilly Matthews by Haslam to the most recent cohort and case-level evidence on algorithmic and AI-mediated delusional content; its integration of phenomenological, cognitive, neurobiological, sociocultural, and forensic frames, which together support a layered rather than merely descriptive reading of pathoplasticity; and its explicit engagement with emerging frontiers, including generative AI, conversational chatbots, and pandemic-era conspiracy narratives, which prior reviews of culture and psychosis have not directly addressed. To our knowledge, it is the first dedicated synthesis of technology-themed delusional content as a distinct clinical topic, and the proposed 5-category typology, the accompanying conceptual framework figure, and the practical recommendations for assessment and treatment offer a working clinical scaffold that can be tested and refined as new technologies emerge.

Limitations of this review include a narrative rather than a systematic methodology, the absence of a formal risk-of-bias assessment, the predominance of Western high-income data [12,44], potential ascertainment bias in retrospective studies [8], and the limited number of large contemporary datasets. The case-level literature self-selects for unusual or pedagogically striking presentations, which means the review overrepresents distinctive content relative to its true clinical prevalence in routine care. The pathoplasticity framework that organizes the synthesis is also vulnerable to a circularity in which any new technological content can be read as confirming the hypothesis; a more falsifiable test would require preregistered prediction of which themes appear and disappear with which technologies, which the existing literature has not undertaken. The review additionally spans historical texts, case reports, small case series, and conceptual papers, which enriches interpretation but limits firm causal inference, and the patient or service-user voice is largely absent from the source material. The tables and conceptual framework are intended as clinically useful syntheses rather than definitive taxonomies.

### Future Research Directions

Several directions for future research are indicated. Prospective cohort studies tracking the evolution of delusional content within individuals over time as technology advances would strengthen the case for pathoplasticity [3,6]. Cross-cultural comparisons are needed to determine whether the patterns observed in Western high-income settings are replicated in low-income and middle-income countries with different trajectories of technological adoption [4,12,44]. Neuroimaging studies comparing technology-themed with nontechnology-themed delusions would clarify whether these presentations represent phenomenologically and neurobiologically distinct subtypes or predominantly surface-level thematic variation [21,37]. Systematic evaluation of targeted interventions, including digital literacy programs for at-risk populations [22,23] and adapted cognitive-behavioral approaches that explicitly engage with patients’ technological explanatory frameworks [7,39], should be prioritized.

### Conclusions

Technology-themed delusions represent a contemporary expression of enduring delusional motifs refracted through the lens of digital culture. Recent single-center clinical data suggest that such content may be common in some contemporary psychosis services, although population prevalence remains uncertain. Clinicians will increasingly need to combine psychiatric expertise with technological literacy and cultural awareness when assessing these presentations. Sustained interdisciplinary research into the evolving relationship between technology, culture, and psychopathology remains both clinically and scientifically important.

## Supplementary material

10.2196/102927 Multimedia Appendix 1 Search strings used in each database.

10.2196/102927 Multimedia Appendix 2 Characteristics of the 43 sources included in the narrative synthesis.

10.2196/102927 Checklist 1 Self-assessment using the SANRA.

## Abbreviations

*DSM-5-TR*: *Diagnostic and Statistical Manual of Mental Disorders*, Fifth Edition, Text Revision
LLM: large language model
PRISMA: Preferred Reporting Items for Systematic Reviews and Meta-Analyses
PROSPERO: International Prospective Register of Systematic Reviews
SANRA: Scale for the Assessment of Narrative Review Articles
