# Supplementary material for: Technology-Themed Persecutory and Related Presentations of Delusions in the Digital Age: Narrative Review
Source: JMIR Ment Health. 2026 Aug 14;13:e102927. doi: 10.2196/102927 (PMC13476003; doi:10.2196/102927)
Supplement: Supplementary file 1 [file mental-v13-e102927-s001.docx]

**Multimedia Appendix 1.** Search strings used in each database.

| **Database** | **Search string** |
| --- | --- |
| **PubMed/MEDLINE** | ("technology delusion"[tiab] OR "technology delusions"[tiab] OR "internet delusion"[tiab] OR "internet delusions"[tiab] OR "internet-related psychosis"[tiab] OR "internet psychosis"[tiab] OR "Truman Show delusion"[tiab] OR "Truman syndrome"[tiab] OR "influencing machine"[tiab] OR "influencing machines"[tiab] OR "microchip delusion"[tiab] OR "microchip delusions"[tiab] OR "cyber-paranoia"[tiab] OR "cyberparanoia"[tiab] OR "electronic chip implant"[tiab] OR "social media psychosis"[tiab] OR "artificial intelligence psychosis"[tiab] OR "AI delusion"[tiab] OR "chatbot delusion"[tiab] OR "large language model psychosis"[tiab]) OR (("internet"[tiab] OR "technology"[tiab] OR "digital"[tiab] OR "cyber"[tiab] OR "social media"[tiab] OR "smartphone"[tiab] OR "algorithm"[tiab] OR "artificial intelligence"[tiab] OR "AI"[tiab] OR "chatbot*"[tiab] OR "large language model*"[tiab] OR "LLM"[tiab] OR "generative AI"[tiab] OR "ChatGPT"[tiab]) AND ("delusion"[tiab] OR "delusions"[tiab] OR "psychosis"[tiab] OR "psychotic"[tiab]) AND ("content"[tiab] OR "cultural"[tiab] OR "pathoplastic"[tiab] OR "historical"[tiab] OR "persecutory"[tiab] OR "case report"[tiab])) |
| **Scopus** | TITLE-ABS-KEY(("technology delusion*" OR "internet delusion*" OR "internet-related psychosis" OR "internet psychosis" OR "Truman Show delusion" OR "influencing machine" OR "microchip delusion" OR "cyber-paranoia" OR "electronic chip implant" OR "social media psychosis" OR "artificial intelligence psychosis" OR "AI delusion" OR "chatbot delusion" OR "large language model psychosis") OR ((internet OR technology OR digital OR cyber OR "social media" OR smartphone OR algorithm* OR "artificial intelligence" OR "AI" OR chatbot* OR "large language model*" OR "LLM" OR "generative AI" OR "ChatGPT") AND (delusion* OR psychosis OR psychotic) AND (content OR cultural OR pathoplastic* OR historical OR persecutor* OR "case report"))) |
| **APA PsycInfo** | (AB("technology delusion*" OR "internet delusion*" OR "internet-related psychosis" OR "internet psychosis" OR "Truman Show delusion" OR "influencing machine" OR "microchip delusion" OR "cyber-paranoia" OR "electronic chip implant" OR "social media psychosis" OR "artificial intelligence psychosis" OR "AI delusion" OR "chatbot delusion" OR "large language model psychosis")) OR (AB(internet OR technology OR digital OR cyber OR "social media" OR smartphone OR algorithm* OR "artificial intelligence" OR "AI" OR chatbot* OR "large language model*" OR "LLM" OR "generative AI" OR "ChatGPT") AND AB(delusion* OR psychosis OR psychotic) AND AB(content OR cultural OR pathoplastic* OR historical OR persecutor* OR "case report")) |

**Note.** A common conceptual search was adapted to each database’s syntax. The search combined a named-phrase block (established terms in the literature, including technology-delusion, internet-delusion, influencing-machine, and AI/LLM-specific phrases) with a concept block built from technology and artificial-intelligence terminology AND psychosis terminology AND a relevance filter, joined by OR. PubMed [tiab] field tagging suppresses MeSH auto-mapping; Scopus TITLE-ABS-KEY tags include Author Keywords; APA PsycInfo AB field searches abstracts. Database searches were complemented by backward citation tracking of the included articles and by targeted location of historical primary sources (e.g., archive.org for the Haslam 1810 and Jay 2004 monographs). Google Scholar was used informally to locate cited works and for backward citation tracking but was not employed as a formal indexed-database search. Title-and-abstract review of database records was conducted as relevance screening for narrative inclusion rather than as a prospectively logged PRISMA-style screening cascade; database hit counts and records-excluded counts were therefore not retained. The final search was run on 1 May 2026.
