# Supplementary material for: Technology-Themed Persecutory and Related Presentations of Delusions in the Digital Age: Narrative Review
Source: JMIR Ment Health. 2026 Aug 14;13:e102927. doi: 10.2196/102927 (PMC13476003; doi:10.2196/102927)
Supplement: Supplementary file 2 [file mental-v13-e102927-s002.docx]

**Multimedia Appendix 2.** Characteristics of the 43 sources included in the narrative synthesis.

| **Ref.** | **Author (Year)** | **Country** | **Study Design** | **Sample** | **Main Contribution** |
| --- | --- | --- | --- | --- | --- |
| 1 | American Psychiatric Association (2022) | – | Diagnostic manual | – | DSM-5-TR diagnostic criteria for delusional disorder; removal of bizarre/non-bizarre requirement |
| 2 | Picardi et al. (2018) | Italy | Cross-sectional | Not reported | Delusional themes across affective and non-affective psychoses; persecutory delusions most prevalent subtype |
| 3 | Sǩodlar et al. (2008) | Slovenia | Retrospective cohort | n = 120 (1881–2000) | Statistically significant increase in technical delusions following radio and television adoption |
| 4 | Stompe et al. (2003) | Austria | Cross-sectional | Multiple countries | Pathoplastic effect on delusional content across cultures |
| 5 | Cannon & Kramer (2012) | USA | Retrospective cohort | Hospital records (1913–1999) | Technology-themed delusions increased across the 20th century; 76% persecutory |
| 6 | Bell et al. (2005) | UK | Case series | n = 4 | Internet influenced delusion aetiology, form, and content; illustrated CBT-based therapeutic approach |
| 7 | Burns et al. (2025) | USA | Retrospective cohort | n = 228 (2016–2024) | 104/201 (51.7%) of those with delusional content described technology delusions; approximately 15% annual increase in odds (odds ratio 1.15); social media and algorithm themes prominent |
| 8 | Gold & Gold (2012) | USA | Case series | n = 5 | Described the Truman Show delusion; proposed the ‘Suspicion System’ model |
| 9 | Pierre (2019) | USA | Conceptual/forensic | – | Integrating non-psychiatric models of delusion-like beliefs into forensic assessment; continuum model |
| 10 | Yang & Crespi (2025) | Canada | Systematic review | Multiple studies | Social media use and disorders of the social brain; mechanisms for delusion-like processes |
| 11 | Ghanem et al. (2023) | UK | Narrative review | Multiple studies | Role of culture on phenomenology of hallucinations and delusions |
| 12 | Baethge et al. (2019) | – | Methodological | – | SANRA scale for quality assessment of narrative review articles |
| 13 | Haslam (1810) | UK | Historical case account | n = 1 | Earliest detailed technology-themed persecution and control account: the ‘air loom’ |
| 14 | Jay (2004) | UK | Historical monograph | – | Comprehensive secondary account of the James Tilly Matthews air loom case |
| 15 | Tausk & Feigenbaum (1992) | Austria | Clinical-theoretical (English translation) | Multiple clinical examples | Seminal formulation of the ‘influencing machine’ phenomenon; described such machines as projections of the body |
| 16 | Tausk (1919) | Austria | Clinical-theoretical (German original) | Multiple clinical examples | Original German-language publication on the influencing machine in schizophrenia |
| 17 | Tan et al. (1997) | Canada | Case report | n = 1 | Early internet-themed persecutory delusion in schizophrenia |
| 18 | Compton (2003) | USA | Case report | n = 1 | Internet-themed delusion case; cultural influence on delusional content |
| 19 | Lerner et al. (2006) | Israel | Case report | n = 2 | Internet delusions as modified persecution and broadcasting delusions |
| 20 | Nitzan et al. (2011) | Israel | Case series | n = 3 | Internet-related psychosis in previously well individuals; loneliness and digital illiteracy as risk factors |
| 21 | Eytan et al. (2002) | Switzerland | Case report | n = 2 | Electronic chip implant delusions proposed as culture-bound syndrome |
| 22 | Hirjak & Fuchs (2010) | Germany | Case series | n = 3 | Phenomenological analysis of ‘technical alien control’ delusions; four properties of modern technology rendering it salient to psychosis |
| 23 | Mason et al. (2014) | UK | Cross-sectional | n = 181 | Developed the Cyber-Paranoia and Fear Scale; cyber-paranoia distinct from general paranoia |
| 24 | Lustig et al. (2021) | UK | Multimodal discourse analysis | YouTube videos | Gangstalking evidence videos; interplay of technology and persecutory belief systems |
| 25 | Yildirim Budak & Yazici Karabulut (2024) | Turkey | Case report | n = 1 | Truman syndrome during online education in adolescent-onset psychosis |
| 26 | Sturm & Albrecht (2021) | – | Review/commentary | – | Contagious conspiracism, 5G, and viral vaccination narratives during COVID-19 |
| 27 | Ahmed et al. (2020) | UK/Spain | Social network analysis | Twitter data | COVID-19 and 5G conspiracy theory propagation on social media |
| 28 | Acar et al. (2022) | Sweden | Cross-sectional | n = 816 | Delusion-proneness trait predicts COVID-19 conspiracy ideation and resistance to belief updating |
| 29 | Acar et al. (2024) | Sweden | Cross-sectional | n = 1,577 | Delusion-proneness predicts COVID-19 vaccination behaviour via conspiracy ideation |
| 30 | Gold & Gold (2015) | USA | Book/conceptual | – | How culture shapes madness; broader cultural context for technology-themed delusions |
| 31 | Freeman et al. (2002) | UK | Theoretical | – | Cognitive model of persecutory delusions |
| 32 | Freeman (2016) | UK | Review | – | Cognitive perspective on understanding and treatment of persecutory delusions |
| 33 | Kapur (2003) | Canada | Theoretical | – | Aberrant salience hypothesis linking dopamine, phenomenology, and pharmacology in schizophrenia |
| 34 | Freeman et al. (2005) | UK | Cross-sectional | n = 1,202 | Hierarchical structure of paranoia in non-clinical population; 15–20% report regular paranoid thinking |
| 35 | Böge et al. (2025) | Germany | Pilot single-arm trial | n = 20 | Feasibility of smartphone-based EMA/EMI for delusions in schizophrenia spectrum disorders |
| 36 | Østergaard (2023) | Denmark | Editorial/viewpoint | – | Commentary on whether generative AI chatbots may generate delusions in psychosis-prone individuals |
| 37 | Buck & Maheux (2026) | USA | Cross-sectional survey | n = 1,003 | Psychosis risk associated with intensive generative AI use; delusion-like experiences in chatbot interactions |
| 38 | Flathers et al. (2026) | USA | Viewpoint/typology | – | Four-part functional typology of LLM-associated psychotic phenomena: catalyst, amplifier, coauthor, object |
| 39 | Stompe et al. (2006) | Multiple | Cross-cultural study | Multiple countries | Pathoplastic effect of culture on psychotic symptoms in schizophrenia across six countries |
| 40 | Chapman et al. (2026) | UK/USA | Conceptual review | – | Reconceptualises the fixity of delusions across the dimensions of conviction, incorrigibility, persistence, and stability; framework for assessing delusional belief |
| 41 | Freeman et al. (2015) | UK | Randomised controlled trial | n = 150 | Reducing worry with cognitive-behavioural therapy reduced persecutory delusions; worry identified as a contributory causal and maintaining factor |
| 42 | Vorontsova et al. (2013) | UK | Cross-sectional | Patients with persecutory delusions | Depression contributes to the maintenance of persecutory delusions; negative affect and negative self-evaluation as maintaining factors |
| 43 | Hudon & Stip (2025) | Canada | Viewpoint | – | Framework conceptualising AI psychosis; sustained anthropomorphic engagement with conversational AI may trigger, amplify, or reshape psychotic experience in vulnerable users |

**Note.** CBT = cognitive-behavioural therapy; EMA/EMI = ecological momentary assessment/ecological momentary intervention; LLM = large language model. Source-type categorisation reflects the primary contribution of each work. Some sources span more than one category (for example, case series that also contain substantial conceptual discussion). ‘–’ denotes not applicable or not reported.
