# Supplementary material for: Technology-Themed Persecutory and Related Presentations of Delusions in the Digital Age: Narrative Review
Source: JMIR Ment Health. 2026 Aug 14;13:e102927. doi: 10.2196/102927 (PMC13476003; doi:10.2196/102927)
Supplement: Supplementary file 3 [file mental-v13-e102927-s003.docx]

**Multimedia Appendix 3.** Self-assessment using the Scale for the Assessment of Narrative Review Articles (SANRA; Baethge et al., 2019).

| **Item** | **Criterion** | **Score** | **Justification** |
| --- | --- | --- | --- |
| **1** | **Justification of the article’s importance for the readership** | 2 | The Introduction explicitly identifies a gap (no prior dedicated synthesis of technology-themed delusional content), outlines clinical relevance (the boundary between plausible privacy concern and delusion is becoming harder to assess), and notes increasing prevalence in contemporary psychosis settings (104/201, 51.7%, of those with delusional content in the Burns et al. cohort). The importance is thoroughly and explicitly justified. |
| **2** | **Statement of concrete aims or formulation of questions** | 2 | Four concrete aims are stated at the end of the Introduction: (1) trace the historical evolution of technology-themed delusional content; (2) characterise its contemporary clinical phenomenology across persecutory and related referential, control, and grandiose themes; (3) examine contributing neurocognitive, emotional, and sociocultural mechanisms; and (4) discuss implications for psychiatric assessment and intervention. These map directly onto the manuscript's section structure. |
| **3** | **Description of the literature search** | 2 | The Methods section specifies three databases (PubMed/MEDLINE, Scopus, APA PsycInfo), reports full search strings in Multimedia Appendix 1, describes eligibility criteria and exclusions, names the screening authors (ME, SAB) and the arbitrating senior author (AAO), provides the final search date (1 May 2026), and reports the source breakdown (43 sources by type) in Multimedia Appendix 2. Search terms and inclusion types are clearly specified. |
| **4** | **Referencing** | 2 | Key statements are consistently supported by references throughout. Historical claims cite primary sources (Haslam, 1810; Tausk, 1919/1992). Empirical claims cite the relevant cohort or case-level data (Burns et al., 2025; Gold and Gold, 2012). Theoretical claims cite cognitive models (Freeman et al., 2002; Kapur, 2003). The DSM-5-TR diagnostic change is cited (reference 1). Referencing is thorough and consistent. |
| **5** | **Scientific reasoning** | 2 | The manuscript incorporates appropriate evidence and consistently identifies study designs in both the text and tables (retrospective cohort, case series, case report, cross-sectional survey, systematic review, viewpoint/editorial). The AI/LLM section is explicitly qualified as based on early commentaries and emerging survey data rather than mature clinical evidence. The gangstalking material is qualified as sociocultural rather than psychiatric evidence. Formal levels-of-evidence terminology is not used, which is standard for psychopathology narrative reviews but represents a potential vulnerability under strict external assessment. |
| **6** | **Appropriate presentation of data** | 2 | Relevant outcome data are generally presented appropriately for the nature of the evidence base. The Burns cohort reports the proportion with technology delusions among those with delusional content (104/201, 51.7%) and the logistic regression result (approximately 15% annual increase in odds; odds ratio 1.15), and the Freeman epidemiological data are reported as a range (15 to 20%). The evidence base is predominantly case-level (9 of 43 sources are case reports or small case series), where confidence intervals and formal effect sizes are not applicable. Where quantitative data are available, the manuscript reports the key metrics accurately. This item is the most vulnerable to downscoring by an external reviewer if confidence intervals for the Burns odds ratio are expected. |

Note. SANRA items are scored 0 (low quality), 1 (intermediate), or 2 (high quality). The sum score ranges from 0 to 12. The extreme values (0 and 2) are used freely to indicate general low or high quality and are not reserved for the worst or best imaginable quality (Baethge et al., 2019). Reference: Baethge C, Goldbeck-Wood S, Mertens S. SANRA—a scale for the quality assessment of narrative review articles. Research Integrity and Peer Review. 2019;4:5.

**Note on review methodology**

This article is a narrative review conducted in line with the Scale for the Assessment of Narrative Review Articles (SANRA) framework. The review was not pre-registered. It is not a systematic review or scoping review and does not aim to comply with PRISMA reporting standards. Records-identified, records-excluded, and full-text-screened counts of the kind reported in PRISMA flow diagrams were not prospectively retained, and we do not present reconstructed counts because such retrospectively assembled numbers can give a false impression of systematic-review rigour. The search strategy is reported in **Multimedia Appendix 1** and the included evidence base is described in **Multimedia Appendix 2**.
